# Role of Small RNAs in Trypanosomatid Infections

**DOI:** 10.3389/fmicb.2016.00367

**Published:** 2016-03-30

**Authors:** Leandra Linhares-Lacerda, Alexandre Morrot

**Affiliations:** ^1^Oswaldo Cruz Foundation, Laboratory on Thymus Research, Institute Oswaldo CruzRio de Janeiro, Brazil; ^2^Department of Immunology, Microbiology Institute, Federal University of Rio de JaneiroRio de Janeiro, Brazil

**Keywords:** small RNAs, microRNAs, host-parasite interaction, trypanosomatid infections, extracellular vesicles, cell communication

## Abstract

Trypanosomatid parasites survive and replicate in the host by using mechanisms that aim to establish a successful infection and ensure parasite survival. Evidence points to microRNAs as new players in the host-parasite interplay. MicroRNAs are small non-coding RNAs that control proteins levels via post-transcriptional gene down-regulation, either within the cells where they were produced or in other cells via intercellular transfer. These microRNAs can be modulated in host cells during infection and are among the growing group of small regulatory RNAs, for which many classes have been described, including the transfer RNA-derived small RNAs. Parasites can either manipulate microRNAs to evade host-driven damage and/or transfer small RNAs to host cells. In this mini-review, we present evidence for the involvement of small RNAs, such as microRNAs, in trypanosomatid infections which lack RNA interference. We highlight both microRNA profile alterations in host cells during those infections and the horizontal transfer of small RNAs and proteins from parasites to the host by membrane-derived extracellular vesicles in a cell communication mechanism.

## Introduction

Trypanosomatid parasites comprise the African trypanosomes (*Trypanosoma brucei*), South american trypanosomes (*Trypanosoma cruzi*) and Leishmania, that profoundly affect mankind and substantially impact world public health (Coura and Viñas, 2010; Alvar et al., 2012). The diseases caused by these parasites predominantly affect the populations of developing regions of Africa, Asia and the Americas; however, population movement creates a new epidemiological challenge with worldwide spreading (Coura and Viñas, 2010; Alvar et al., 2012). Through different mechanisms these parasites establish a successful infection. Among these mechanisms, the small RNAs emerge as new players in the host-parasite interplay.

Small non-coding RNAs play an essential regulatory role in complex biological systems without protein translation (Aalto and Pasquinelli, 2012). Of these RNAs, the short-length RNA molecules (ranging from 20 to 30 nucleotides), such as microRNAs, small interfering RNAs (siRNAs) and Piwi-interacting RNAs (piRNAs), stand out. Through sequence complementarity, these RNAs guide recognition of target genes within the ribonucleoprotein (RNP) complex and typically reduce the expression of a specific gene through the process of RNA interference (RNAi). Despite having the same mechanism of action, these different classes of regulatory RNAs differ in their association with Argonaute (AGO) protein family members to form the RNP complex, in their biogenesis, in their gene regulation pathways and in their biological functions (Ghildiyal and Zamore, 2009). Furthermore, the world of small non-coding RNAs is expanding, with new classes continuing to be discovered, even in organisms that were not previously thought to express small RNA-mediated pathways, such as *T. cruzi* and some *Leishmania* species (Ullu et al., 2004; Ghildiyal and Zamore, 2009; Garcia-Silva et al., 2010a,b; Lye et al., 2010).

RNA-mediated silencing is an evolutionarily conserved mechanism that may have evolved together with parasite infection, as parasites developed strategies to interfere with host microRNA populations, thus recognizing the RNAi pathway as a new means of reshaping their environment to evade host immune surveillance and establish a successful infection (Cerutti and Casas-Mollano, 2006; Hakimi and Cannella, 2011). Obviously, changes in microRNA profiles might also be a defense mechanism of the infected cell. Nevertheless, the alteration of host microRNA levels after parasitic infection has been demonstrated (Geraci et al., 2015; Linhares-Lacerda et al., 2015), with some data revealing the intricate connection between the parasite and the RNAi machinery of the host organism (Ghosh et al., 2013). Moreover, the identification of predictive microRNA signatures associated with each specific parasitic infection could aid in the development of tools for diagnosis, prognosis, monitoring therapy and improving patient stratifications (Manzano-román and Siles-lucas, 2012).

In this mini-review, we briefly discuss current knowledge about the involvement of small RNAs in host-parasite interactions on trypanosomatid parasites that lack the AGO and Dicer genes and as a consequence do not have functional RNAi machinery. These parasites include *T. cruzi* (the etiologic agent of Chagas disease), *Leishmania major* and *Leishmania donovani* (which cause cutaneous leishmaniasis and visceral leishmaniasis, respectively). We focus on the microRNA profile alterations that occur in host cells due to infection with those parasites and on the trans-kingdom transfer of small RNAs and proteins from parasites to the host by membrane-derived extracellular vesicles (EVs) in a cell communication mechanism that may favor parasite survival.

## MicroRNA profile modulation due to parasitic infection

The elaborate relationship between parasites and their hosts aims to establish a successful parasite infection/infestation and promote survival, with parasites manipulating the host cellular machinery to avoid and regulate the host immune effector response (Manzano-román and Siles-lucas, 2012). In this context, gene expression modulation by microRNAs may be an ideal tool for parasites because microRNAs can function as master switches of many biological functions, fine tuning protein production (Zheng et al., 2013). It is reasonable to propose that cellular infection will be counteracted by cellular microRNAs that target crucial host factors as a defense mechanism; however, parasites subvert microRNA-directed functions as a means of altering gene expression in host cells (Hakimi and Cannella, 2011).

### MicroRNAs are related to cardiac alterations and thymic atrophy in chagas disease

The alteration of host microRNA levels after *T. cruzi* infection has been demonstrated in a murine model (Linhares-Lacerda et al., 2015; Navarro et al., 2015) and in Chagas disease patients (Ferreira et al., 2014). Chagas disease is a neglected tropical illness that is endemic to Latin America (Coura and Borges-pereira, 2012) and has an acute phase characterized by bloodstream circulating parasites and tissue parasitism, in addition to an intense immune response and hormonal imbalance (Pérez et al., 2011). Immune effector responses control *T. cruzi* numbers in the blood, and individuals enter the chronic phase of the disease with low parasites levels in several tissues (de Meis et al., 2013). Moreover, chronic infection can persist undetected, but ~30% of patients develop severe complications, such as abnormal heart rhythm, heart failure, and digestive problems (Clayton, 2010; World Health Organization, 2010a).

The hearts of mice with experimental acute *T. cruzi* infection present an intense inflammatory cell infiltrate with myocarditis, arrhythmia and parasite nests in addition to a modified microRNA expression profile. Upon infection, 113 of 641 microRNAs were dysregulated; moreover, some microRNAs correlated with the maximal heart rate-corrected QT interval, which is a cardiac alteration (Navarro et al., 2015). Resembling the experimental model, chronic Chagas disease patients who develop cardiomyopathy can also present alterations in heart microRNAs and heart arrhythmia, among other cardiac complications. miR-1, miR-133a-2, miR-133b, miR-208a, and miR-208b were significantly downregulated in Chagas disease patients in comparison to uninfected patients (heart transplant donors). Moreover, in a comparison between two cardiomyopathy groups (chronic Chagas disease patients and dilated cardiomyopathy patients), miR-1, miR-133a-2, and miR-208b expression was reduced in infected patients (Ferreira et al., 2014). These microRNAs are highly enriched in the heart, where they regulate heart development and myocyte differentiation (Lagos-Quintana et al., 2002; Chen et al., 2006). In addition, atypical expression of these microRNAs has been linked to cardiovascular diseases (Carè et al., 2007; Ikeda et al., 2013). Therefore, variations of host microRNAs in experimental models of acute Chagas disease could shed light on the mechanism that triggers heart clinical alterations, with possible relevance for chronic Chagas disease patients with cardiomyopathy, as the downregulation of miR-208 was detected in both patients and mice infected by *T. cruzi* (Ferreira et al., 2014; Navarro et al., 2015). It is noteworthy that cardiac damage releases miR-208 and other factors into the bloodstream, and the levels of these factors exhibit distinctive patterns that correlate with different cardiovascular diseases, showing great potential for use as biomarkers for cardiac illness (Gupta et al., 2010). However, no data are available concerning circulating microRNAs in chronic Chagas disease patients who develop cardiomyopathy.

**Figure 1:**
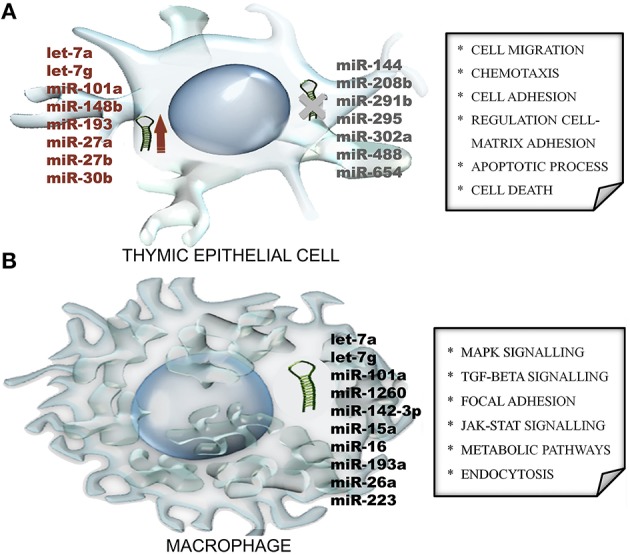
**MicroRNA modulation during parasitic infection: clues from Gene Ontology (GO) enrichment analysis of microRNA ***in silico***-predict targets. (A)** Mice with acute *Trypanosoma cruzi* infection modulate microRNA expression in thymic epithelial cells, with some microRNAs upregulated (red) and others undetectable (gray). The regulated microRNAs were used to identify biological processes (depicted within the square) that were over-represented among the list of microRNA target genes. **(B)** The examination of mature microRNA expression patterns in human macrophages revealed unique microRNA profiles in response to the *Leishmania major* and *L. donovani* parasites. In addition, biological pathway enrichment was performed for the dysregulated microRNAs to identify signaling pathways (depicted within the square) that might be involved.

In addition to heart manifestations, mice with acute *T. cruzi* infection also present a severe thymic atrophy, primarily due to the apoptosis of CD4^+^CD8^+^ double-positive immature T cells and also due to migratory abnormalities that release potential autoreactive T cells to secondary lymphoid organs, which may play a role in the chronic phase of the disease (Savino, 2006). The development of increased T cell migration may be a consequence of signals delivered by thymic epithelial cells (TECs) that enhance the deposition of extracellular matrix proteins upon infection (Cotta-de-Almeida et al., 2003; Pérez et al., 2012). These signals might be under the control of microRNAs that are modulated in TECs from infected mice before the induction of thymic atrophy. Interestingly, microRNAs were primarily upregulated (29 out of 85 microRNAs), even if the TECs that were sorted from the thymus exhibited a cortical or medullary phenotype. Furthermore, Gene Ontology (GO) enrichment analysis of microRNA targets was used to identify biological processes that were over-represented among the list of target genes (Figure 1A). Indeed, the theoretical relationships of these microRNAs with their putative RNA targets revealed transforming growth factor-β (TGF-β) as a molecular node of infection, as the gene encoding its receptor (the Tgfbr1 gene) appears in the middle of our microRNA network, with 8 different microRNAs (let-7a, let-7g, miR-101a, miR-148b, miR-193, miR-27a, miR-27b, and miR-30b) regulating this gene (Linhares-Lacerda et al., 2015).

Taken together, these reports highlight the importance of microRNA alterations in Chagas disease. Furthermore, additional studies are needed to define microRNA biomarkers of *T. cruzi* infection. In this context, it is reasonable to hypothesize that miR-208 is a potential biomarker for *T. cruzi* infection because this microRNA was downregulated in both the human and mouse heart and undetectable in TECs from *T. cruzi*-infected samples, revealing a possible common regulation pattern in response to *T. cruzi* infection.

### Macrophages change their microRNA profiles in response to *Leishmania*

Sophisticated strategies for surviving and establishing a successful infection, such as antigen presentation inhibition, were developed by *Leishmania* parasites, which cause cutaneous or visceral diseases and are among the neglected diseases (World Health Organization, 2010b). *Leishmania* is an obligate intracellular pathogen in mammalian hosts and primarily infects macrophages, where it avoids anti-parasitic responses and subverts host innate immunity. The parasite modifies both microRNAs and mRNAs from the host, leading to altered expression of lipid metabolic genes, among other genes, resulting in reduced cholesterol synthesis, the disruption of membrane lipid rafts and the inhibition of antigen presentation to T cells (Ghosh et al., 2013; Chakraborty et al., 2015).

Upon infection with *L. major*, human primary macrophages change the microRNA-levels of 64 of 365 microRNAs, as assessed via a quantitative PCR time course. These dysregulated microRNAs virtually targeted several transcripts with critical cellular functions, such as cellular movement, secretion, communication, enzyme production, activity in the extracellular space, signal transduction, and gene expression naturally induced by an abiotic stimulus, which were all evaluated via a GO enrichment analysis followed by a pathway analysis (Lemaire et al., 2013). Additional examination of mature microRNA expression patterns in *L. major*- and *L. donovani*-infected human primary dendritic cells and macrophages using next-generation sequencing revealed unique mature microRNA expression profiles in response to both parasite species in different human host cell types. Indeed, *L. donovani*-infected cells exhibited higher expression of the identified microRNAs than *L. major*-infected cells. The biological pathway enrichment was performed again with predicted targets of the dysregulated microRNAs and identified the mitogen-activated protein kinase (MAPK) signaling pathway, among others (Figure 1B), regardless cell type or the infecting *Leishmania* species (Geraci et al., 2015).

In general, those studies revealed the remarkable capacity of *Leishmania* to modify microRNA expression in the host; nevertheless, the biological significance of the dysregulated microRNAs requires further investigation. For this purpose, the use of microRNA mimics and inhibitors is an excellent tool. For example, the transfection of the mmu-miR-210-5p inhibitor into *L. major*-infected murine macrophages significantly decreased the infection rates of these cells, suggesting a role of miR-210 in anti-parasitic activity (Frank et al., 2015). Moreover, the RNA targets obtained via *in silico* prediction require experimental evidence with further functional analysis to determine the role of each microRNA/mRNA-target in the specific pathways in which it participates. In the future, the knowledge gained from those investigations will assist in the discovery of new targets for diagnostics or therapeutic approaches.

## Trans-kingdom transfer of extracellular vesicles

Cells exchange information with their environments, influencing the behavior of other cells, and themselves. Cells can communicate through a variety of chemical, mechanical, and biological signals that trigger cell signaling and allow the cells to process information from the outside to support survival. Intercellular communication through biological signals involves the transfer of many different molecules, such as hormones, cytokines, and small RNAs, primarily via membrane vesicle trafficking (Barteneva et al., 2013). Taking advantage of cellular communication through the transfer of membrane-derived extracellular vesicle (EV) cargo to host cells, parasites manipulate host functions to establish a successful infection (Marcilla et al., 2014). In this section, we review the trans-kingdom transfer of EVs from *T. cruzi* and *L. donovani* to host cells.

### Trypanosomatid parasites deliver small RNAs through extracellular vesicles to host cells

**Figure 2:**
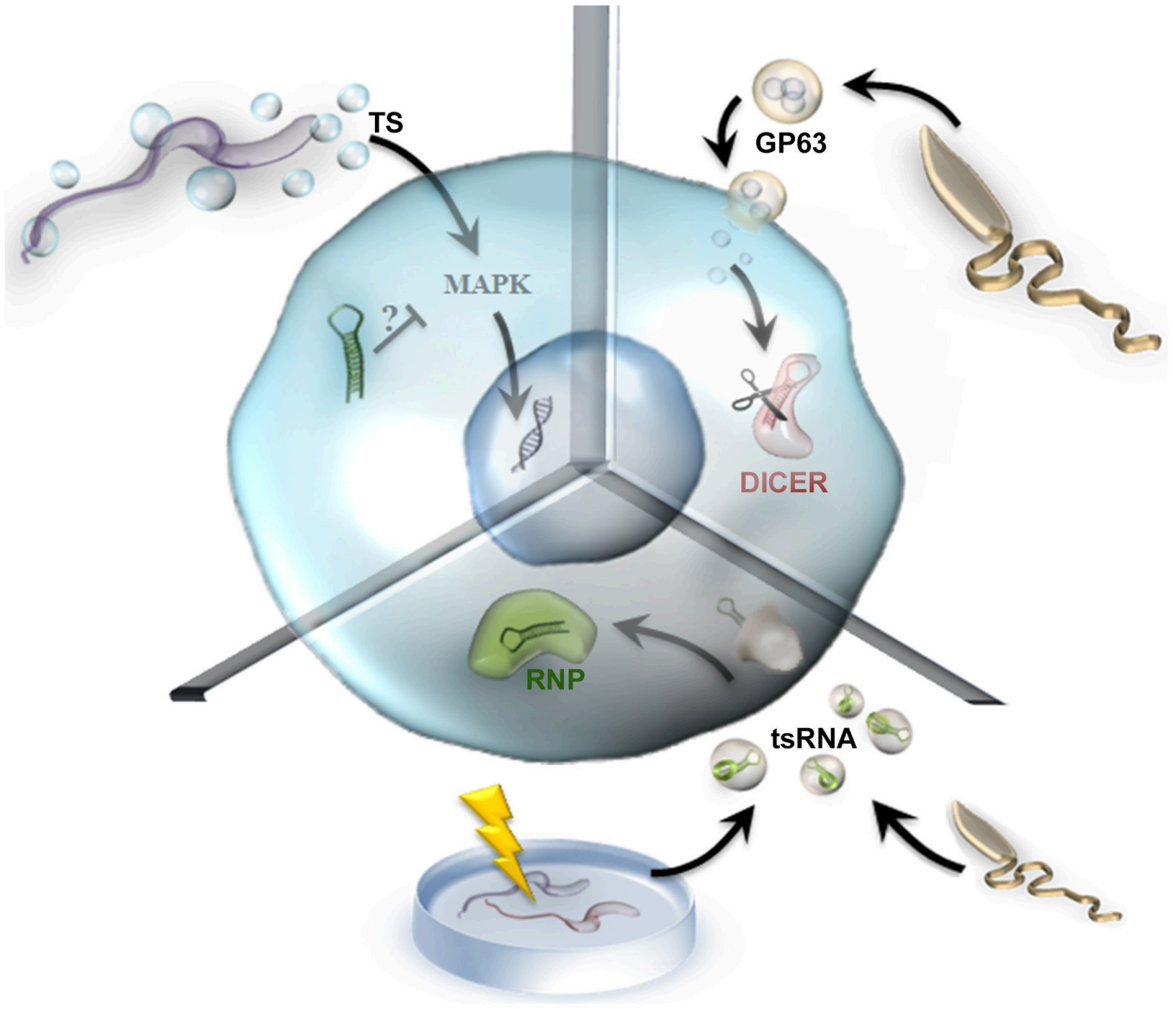
**Transfer of parasitic extracellular vesicle cargo to host cells**. In this figure, we used a generic host cell to exemplify three different molecules delivered by parasitic extracellular vesicles. In the **upper left part** of the diagram, *Trypanosoma cruzi* releases EVs containing TS (trans-sialidase, *blue circles*) that can trigger gene modulation through the MAPK signaling pathway, which may be regulated by microRNAs. In the **upper right part** of the diagram, *Leishmania donovani* releases GP63 vesicles (*orange circles*) that cleave DICER (*red*), impairing microRNA maturation. Finally, in the **bottom part** of the diagram, EVs containing tsRNA (tRNA-derived small RNAs, in *green*) from Leishmania and from stressed *Trypanosoma cruzi* parasites modulate gene expression and might form RNP complexes (*green*).

EVs are key players in cell-to-cell communication. Like other pathogens, *T. cruzi* releases proteins associated with vesicles into the extracellular milieu to enable pathogen survival and replication within the host (Marcilla et al., 2014). *T. cruzi*'s protein vesicle content was defined in a proteomic study that among other classes, identified a relatively high proportion of RNA-binding proteins, suggesting a possible role in intercellular communication and gene expression regulation (Bayer-Santos et al., 2013). On the other hand, short transcriptome analysis using unbiased and genome-wide deep sequencing indicated an abundance of small RNAs derived from non-coding RNAs, of which tRNA-derived small RNAs (tsRNAs) derived from the 3′end with a median length of 38 nt were the most frequently detected type. Moreover, a comparison between certain tRNA isoacceptors from which tsRNAs were derived revealed that tsRNAs are differentially expressed and may be actively produced rather than being random degradation products from tRNA turnover (Franzén et al., 2011). Quite strikingly, *T. cruzi* lacks functional RNAi machinery but does express a unique open reading frame for an AGO/PIWI protein with the conserved domain architecture of a canonical AGO in all stages of its life cycle (Garcia-Silva et al., 2010b). Interestingly, tsRNA colocalizes with this distinctive trypanosomatid AGO protein (TcPIWI-tryp) in EVs that are transferred to surrounding parasites and to susceptible mammalian host cells, where the protein changes gene expression profiles (Garcia-Silva et al., 2010a, 2014a,b; Figure 2).

Recently, evidences demonstrated that Leishmania parasites release exosomes containing RNA sequences and that this exosomos and their cargo can be internalized by host cells. This is true for two different species of Leishmania, namely *L. donovani* and *L. braziliensis*, suggesting that the packing of specific RNA sequences into exosomes may be a conserved phenomenon in Leishmania (Lambertz et al., 2015). Like *T. cruzi* the authors found a richness of tRNA fragments originating from a small subset of tRNA isoacceptors, the tsRNAs, in both species, moreover in *L. braziliensis* which is RNAi-competent organism, they also found sequences derived from siRNA-coding regions in both sense and anti-sense suggesting that they appear as double-stranded RNAs in exosomes (Lambertz et al., 2015) (Figure 2). Although these finds, the *in vivo* biological effect of EVs carrying tsRNAs remains obscure, and more studies are required to assess the molecular mechanisms associated with these non-coding RNAs.

*T. cruzi* also releases EVs containing members of the trans-sialidase glycoprotein superfamily. One member of this family led to the aggravation of experimental Chagas disease, with a severe inflammatory reaction of the heart and an increased number of amastigote nests in animals that received these EVs prior to *T. cruzi* infection (Trocoli Torrecilhas et al., 2009). The *T. cruzi* trans-sialidase transfers host sialic acid to parasite surface glycoconjugates, a process that supports host–cell recognition, infectivity, and parasite survival. Indeed, this trans-sialidase activity can remodel parasite glycomolecules, altering host immune responses against the parasite and playing a role as a virulence factor (Freire-de-Lima et al., 2012).

The presence of the trans-sialidase was confirmed in the peripheral blood of chronic Chagas disease patients, where the antibody titre against the trans-sialidase increased with the frequency of peripheral double-positive immature T cells, potentially contributing to the clinical manifestations observed in the chronic phase of the disease. On the other hand, the thymus of *T. cruzi*-infected mice presents trans-sialidase depots near the parasite nests, which play a role in thymic atrophy and the premature release of double-positive CD4^+^CD8^+^ immature T cells. In contrast, intrathymic trans-sialidase injection increased the splenic double-positive immature T cell population and activated the MAPK/JNK signaling pathway in immature T cells (Nardy et al., 2013). Despite great advances toward understanding the effects of the *T. cruzi* trans-sialidase (Alves and Colli, 2010), the components involved in this signaling process remain a mystery. This process appears to be cell type-dependent, with MAPK/ERK-1/2 induction in naive splenic CD4 T cells (Todeschini et al., 2015), MAPK/JNK induction in immature T cells (Freire-de-Lima et al., 2012) and NF–kB induction in endothelial cells (Dias et al., 2008), but no microRNAs have been described to date. We suggest miR-199a as a good candidate for future studies of the *T. cruzi* trans-sialidase pathway. miR-199a regulates the PI3K/Akt and ERK/MAPK signaling pathways (Santhakumar et al., 2010) and targets a sialyltransferase (ST6 β-galactosamide α-2,6-sialyltransferase 1, ST6GAL1) (Minami et al., 2013). In addition, miR-199a is upregulated during human hypertrophy-related heart failure (van Rooij et al., 2006); however, we do not know how the expression pattern of miR-199a changes in the hearts of Chagas disease patients (Figure 2).

Taken together, the EVs from *T. cruzi* could be an additional strategy for modulating host cells via pathogen-to-host communication through the delivery of tsRNAs and virulence factors. However, the involvement of small RNAs is a recent discovery, and more studies are needed to elucidate this issue.

### Exosome cargo impairs microRNA maturation during *Leishmania donovani* infection

During intercellular signaling and communication, EVs are used as a mechanism to actively regulate protein release from the cell. In *Leishmania*, changes in parasite culture temperature (26/37°C) lead to protein-specific enrichment in vesicles, affecting the cargo of the released exosomes. This exosome-based protein secretion mechanism delivers cargo to macrophages and triggers biological effects, such as the induction of interleukin-8 secretion (Silverman et al., 2010). Thus, EVs serve as an excellent pathogen-to-host communication process that could deliver effector molecules, such as proteins, and may also release RNAs into the host cytosol.

In this context, the delivery of the *Leishmania* surface protein metalloprotease GP63, which is a membrane-bound glycosylphosphatidylinositol (GPI)-anchored glycoprotein and a known virulence factor (Brittingham et al., 1995), participates in the parasite's strategy to evade immune surveillance. *L. donovani* extracts membrane cholesterol from macrophages, preventing T cell stimulation and causing hypocholesterolaemia that leads to protection against this infection, with an inverse correlation between serum cholesterol levels and parasite load in infected mice. In fact, the delivery of exosomes containing GP63 produced by Kupffer cell-resident parasites to hepatocytes impairs miR-122 activity by cleaving DICER1, which is a primary target of GP63 (Ghosh et al., 2012, 2013; Figure 2). DICER1 processes pre-microRNAs into mature microRNAs and transfers those microRNAs to AGO, forming the RNP complex. In the presence of GP63, hepatocytes accumulated pre-miR-122 and failed to form the RNP-miR-122 complex, possibly leading to the downregulation of cholesterol synthesis because miR-122 is responsible for lipid metabolism and liver homeostasis (Girard et al., 2008; Ghosh et al., 2013). Interestingly, the restoration of Dicer1 expression in parasite-infected livers increased miR-122 expression and restored serum cholesterol levels, with a drastic reduction in liver parasite load. Therefore, this process is a sophisticated example of how parasites evolved strategies to combat regulatory RNA functions in host cells, leading to an important metabolic change that promotes pathogenesis.

## Concluding remarks

Although few publications are available on this topic, current knowledge emphasizes the alteration of microRNA profiles during infection and EV cargo delivery during host interactions with the parasites *T. cruzi, L. major*, and *L. donovani*, which lack functional RNAi machinery. In this mini review, we highlighted some interesting findings in these fields and raised questions for further investigation, such as the status of miR-208 as a potential biomarker for *T. cruzi* infection, the presence of tsRNA in *Leishmania* EVs and the involvement of microRNAs in trans-sialidase triggered pathways during *T. cruzi* infection.

The main open question is to determine the role of each microRNA/mRNA-target in specific pathways through functional analysis and to investigate the importance of these factors in pathogenesis. The knowledge acquired from these futures studies will be useful for aiding the discovery of new targets for diagnosis or therapeutic approaches.

## Author contributions

All authors listed, have made substantial, direct and intellectual contribution to the work, and approved it for publication.

### Conflict of interest statement

The authors declare that the research was conducted in the absence of any commercial or financial relationships that could be construed as a potential conflict of interest.
